# Pathogenesis of glucocorticoid-induced avascular necrosis: A microarray analysis of gene expression *in vitro*

**DOI:** 10.3892/ijmm.2015.2273

**Published:** 2015-07-06

**Authors:** YANYAN BIAN, WENWEI QIAN, HONGLING LI, ROBERT CHUNHUA ZHAO, WANG XING SHAN, XISHENG WENG

**Affiliations:** 1 Department of Orthopedics, Peking Union Medical College Hospital, Chinese Academy of Medical Sciences and Peking Union Medical College, Beijing 100730, P.R. China; 2 Institute of Basic Medical Science, Chinese Academy of Medical Sciences, Beijing 100730, P.R. China

**Keywords:** microRNA, glucocorticoid, mesenchymal stem cells

## Abstract

Avascular necrosis of the femoral head (ANFH) occurs following exposure to corticosteroids, and the proliferative capacity of the mesenchymal stem cells (MSCs) belonging to ANFH was reduced. The previous studies indicate that microRNA (miRNA) has an important regulatory role during proliferation and osteogenic differentiation of MSCs. Therefore, MSCs were obtained from healthy adults, and were cultured and osteogenically-induced by different dexamethasone concentrations. The proliferation and osteogenic differentiation capacities were examined through observing cellular morphology, alkaline phosphatase and alizarin red; miRNA expression was investigated using an miRNA gene chip and miRNA of differential expressions were retrieved through a database to analyze its regulatory effect. Dexamethasone at a concentration of 10^−7^ mol/l induced the proliferation and osteogenic differentiation of MSCs and resulted in evident miRNA expression profile changes. In total, 11 miRNAs were upregulated at 10^−7^ mol/l while 6 were downregulated, and partial miRNA was identified to participate in the regulation of cell proliferation and cell apoptosis, MSC osteogenic differentiation, lipid metabolism and other processes.

## Introduction

MicroRNAs (miRNAs or miRs) are small, single-stranded RNA that are 18–25 nucleotides in length. They regulate gene expression post-transcriptionally, by inhibiting translation or degrading mRNA, based on the complementarity base-pairing between the miRNA and the target mRNA. miRNAs contribute to a variety of biological processes, including proliferation, development, differentiation, apoptosis, metabolism and cancer development (1–4). The tissue specificity and temporal expression of miRNAs provide a basis for them as diagnostic and prognostic markers of disease, which has been confirmed in cancer research (5–7).

Previous studies have indicated that miRNAs have an important regulatory role during the osteogenic differentiation of mesenchymal stem cells (MSCs). Gao *et al* (8) analyzed the miRNA expression profile during the differentiation of human MSCs and identified that 4 miRNAs were downregulated and 3 were upregulated. Using a bioinformatics analysis, the investigators found that the corresponding target genes of the miRNAs downregulated during osteogenic differentiation, i.e., *miRNA-31*, *miRNA-106a* and *miRNA-148a*, were *RUNX2*, *CBFB* and *BMP*. Additionally, Zeng *et al* (9) identified that *miRNA-100* affected the differentiation of stem cells towards osteogenesis by regulating its target gene, *BMPR2*. Eskildsen *et al* (10) reported that *miRNA-138* prevented the osteogenic differentiation of MSCs.

During steroid-induced femoral head necrosis, the proliferative capacity (11) and osteogenic differentiation capacity of the MSCs of the femoral head are reduced (12). As observed in *in vivo* experiments, while low doses of glucocorticoids are required to induce the osteogenic differentiation of stem cells, high-doses of glucocorticoids prevented proliferation and osteogenic differentiation (13,14) through glucocorticoid receptor (GR) and AP-1; the ectopic expression of *RUNX2* could partially reverse these effects (15).

While the effects of glucocorticoids and miRNAs on MSCs have already been reported, to the best of our knowledge, there have been no definite reports on whether glucocorticoids can alter the expression of miRNAs during the differentiation of MSCs, thus regulating osteogenic differentiation at the post-transcriptional level.

## Materials and methods

### Ethics statement

All the procedures followed were in accordance with the ethical standards of the responsible committee on human experimentation (Peking Union Medical College Hospital Ethics Committee, Beijing, China) and with the Helsinki Declaration of 1975, as revised in 2000. Informed consent was obtained from all the patients included in the study.

### Separation and culture of human MSCs

The marrow samples in the study were obtained from the 3 patients who underwent surgery for total hip arthroplasty due to hip osteoarthritis.

The separation of MSCs in the myeloid tissue was performed as described by Pittenger *et al* (16). A sterile marrow puncture needle and a 10-ml syringe were used to extract myeloid tissue from the side of the femur. The sample was transferred into a test tube with culture medium and heparin (4,000 U/ml) for anticoagulation, diluted with an equal volume of phosphate-buffered saline (PBS) and mixed. The mixture was allowed to stand for 30 sec, after which the sediment was discarded and an equal quantity of 1.077 g/ml lymphocyte-separating medium (Mediatech, Herndon, VA, USA) was added. The samples were centrifuged at 778 × g for 20 min, and the monocytes that were present at the white middle layer were carefully collected. The monocytes were washed with 10 ml D-Hank's solution and collected following centrifugation at 280 × g for 6 min. The cells were resuspended at a density of 2×10^6^ cells/ml in nutrient solution containing 58% Dulbecco's modified Eagle's medium (DMEM)/F12, 40% MCDB-201, 2% fetal calf serum, 10 ng/ml EGF, 10 ng/ml PDGF, 1X insulin-transferrin-selenium, 1X linoleic acid-bovine serum albumin (BSA), 50 *µ*M β-mercaptoethanol, 2 mM L-glutamine, 100 *µ*g/ml penicillin and 100 U/ml streptomycin sulfate. After 2 days, the cells that were not attached were discarded and half the growth media was replaced every 3 days. When the cells reached 70–80% confluence, they were detached using 0.25% trypsin and 0.01% EDTA, and were reseeded at a ratio of 1:3.

### Immunophenotyping of cells

Following the digestion of the MSCs with pancreatin, the cells were resuspended in PBS with 0.5% BSA. Primary antibodies were added and the samples were incubated at 4°C for 30 min. The following primary monoclonal mouse anti-human antibodies were used: Cluster of differentiation 29 (CD29, mouse, Cat. no. 555442; BD Biosciences), CD34 (mouse, Cat. no. 550760, BD Biosciences), CD44 (mouse, Cat. no. 550988; BD Biosciences), CD105 (mouse, Cat. no. M3527; DAKO), HLA-DR (mouse, Cat. no. 555810; BD Biosciences) and Flk-1 (mouse, Cat. no. sc-6251; Santa Cruz Biotechnologies). To detect the intracellular antigen Flk-1, the cells were fixed in 1% paraformaldehyde at 4°C for 15 min prior to incubation with the primary antibody and were dried with 0.1% escin at room temperature. An immunoglobulin G antibody of a similar isotype was used as the negative control. Subsequent to washing the cells with PBS, the secondary antibody conjugated with fluorescein isothiocyanate was added, and the samples were incubated at 4°C for 30 min. Finally, the cells were washed twice, resuspended in 500 *µ*l PBS and analyzed by flow cytometry (FCM).

### Induction of differentiation

Third-generation MSCs were seeded at a cellular density of 2×10^4^ cells/cm^2^ in 25-cm^2^ (T25) culture flasks for RNA extraction or in 24-well plates for staining. The cells were allowed to adhere overnight. When they reached 70 or 80% confluence, the osteogenic induction culture solution (10% FBS, 10 nM dexamethasone, 0.2 mM ascorbic acid and 10 mM sodium β-glycerophosphate in H-DMEM culture medium) was replaced with 10^−7^ or 10^−9^ mol/l of dexamethasone, respectively. Half the solution was replaced every 2 days. The cells grown in the T25 culture flasks were collected 0, 6 and 12 days after the induction of differentiation. RNA was extracted from the cells, and the expression of osteogenic phenotype marker genes were measured by quantitative polymerase chain reaction (PCR). On days 6 and 12, the calcification and mineralization matrix of the differentiated cells in the 24-well plates were assessed by alkaline phosphatase and alizarin red.

Additionally, third-generation MSCs were seeded at a cellular density of 2×10^4^ cells/cm^2^ in a T25 culture flask, allowed to adhere overnight and grown to 70 or 80% confluence. After 48 h of stimulation with 10^−7^ and 10^−9^ mol/l dexamethasone, respectively, the cells were lysed in TRIzol for RNA extraction. The extracted RNA was sequenced.

### Cellular staining

For alkaline phosphatase staining (adopt kit; Tianjin Blood Research Institute, Chinese Academy of Medical Sciences), a droplet of no. 1 liquid was added to each sample in a 24-well plate and the samples were incubated at room temperature for 1 min. The samples were subsequently rinsed with running water for 2 min and dried. Action liquid was added, and the samples were incubated at 37°C for 2 h, following which they were washed under running water for 2 min. No. 5 liquid was added and the samples were incubated for 5 min, following which they were washed under running water for 2 min and dried. For alizarin red staining, the 24-well plates were washed twice in PBS, fixed with 95% ethanol, washed with double-distilled water three times, and subsequently, 0.1% alizarin red-Tris-HCl (pH 8.3) was added. The samples were incubated at 37°C for 30 min, following which they were washed with distilled water, dried and sealed.

### RNA extraction

RNA was extracted from the cells according to the manufacturer's instructions for extracting total RNA using the TRIzol™ reagent nucleic acid separation kit (Gibco/BRL, Grand Island, NY, USA). The optical density values of the RNA sample were measured at 260 and 280 nm using a NanoDrop biological spectrophotometer to determine the concentration of the DNA sample and to assess the quality of the sample. The A_260_/A_280_ value of the purified RNA sample should be 1.8–2.0. A value >2.0 indicates that the RNA is contaminated; a value <1.8 indicates that the phenol or proteins were not completely eliminated from the sample.

### Extraction of small RNA and construction of the cDNA library

Following mixing, 2 *µ*l SRA Ladder and 2 *µ*l SRA Gel Loading dye (Illumina, Inc., San Diego, CA, USA) were placed in a 200-*µ*l centrifuge tube, heated for 65°C for 5 min, and centrifuged at 18,000 × g at room temperature for 10 sec after cooling on ice. The SRA ladder and the sample RNA were loaded onto the same gel and electrophoresed for 1 h at 200 V, following which the gel was placed into a sterile chamber and stained with TBE/ethidium bromide for 2 min. The gel was visualized using a UV transilluminator. According to the ladder markings, the portion of the gel corresponding to RNA with a length of 18–30 nucleotides was excised and placed into a microcentrifuge tube. The sample was centrifuged at 20,000 × g at roomtemperature for 2 min, mixed with 300 *µ*l 0.3 mol/l sodium chloride solution and incubated at room temperature for 4 h to elute the RNA. The sample was subsequently transferred to a Spin X cellulose acetate filtration column and was centrifuged at 20,000 × g at room temperature for 2 min. Following this, l *µ*l glycogen and 750 *µ*l absolute ethyl alcohol at room temperature were added to the Spin X filtrate, and the RNA was allowed to precipitate at −80°C for 30 min. The sample was centrifuged at 20,000 × g at 4°C for 25 min, following which the supernatant was removed and the RNA pellet was washed with 750 *µ*l 75% ethanol at room temperature. The RNA pellet was dried and resuspended in 5.7 *µ*l RNase-free water.

The small RNA that was isolated was ligated at the 5′ and 3′ terminals and mixed with 0.5 *µ*l SRA for reverse transcription. The sample was heated at 65°C for 10 min and cooled, and 2 *µ*l 5X first-strand buffer solution, 0.5 *µ*l 12.5 mM dNTP mix, 1 *µ*l 100 mM dithiothreitol and 0.5 *µ*l RNA enzyme inhibitor were added. The sample was heated at 48°C for 3 min in a thermal cycler, and 1 *µ*l SuperScript II reverse transcriptase was added. The sample was incubated at 44°C for 1 h in a thermal cycler. Finally, 40 *µ*l PCR Master mix was added to 10 *µ*l single-stranded reverse transcription cDNA.

### miRNA high-throughput sequencing

The standard procedures for the Illumina HiSeq 2000 sequencing platform were performed.

### Bioinformatics analysis

The data from 6 samples were obtained through high-throughput sequencing and were subjected to further analysis. The Burros-Wheeler Aligner software was used to exclude rRNA, tRNA and other non-coding RNAs, and the remaining sequences were cross-referenced to the Blast database to identify known miRNA genes. The miRBase 18.0 database and PatScan were used to compare the data and generate statistics for the trusted platform module (TPM) values of the mature miRNAs. These data represent the relative expression of the miRNAs and are obtained by dividing the total sequencing number by an absolute expression quantity by 100,000 times.

The 6 samples were divided into 3 groups, representing the control MSCs and the cells treated with 10^−7^ and 10^−9^ mol/l dexamethasone. The TPM values of the 3 conditions were compared. P-values were calculated using the χ^2^ method, and P-values <0.05 were considered to indicate a statistically significant difference. As such, miRNA expression differences resulting from a high concentration of dexamethasone (10^−7^ mol/l) and a physiological concentration of dexamethasone (10^−9^ mol/l) were obtained. The miRNA expression pattern differed among the 3 groups, and candidate miRNAs that were differentially regulated among individuals were also excluded.

Differentially expressed candidate miRNA were screened using online software databases, miRBase, miRDB, TargetScan and PicTar, to analyze the possible target sites of the miRNAs and their possible regulatory effects.

## Results

### Characteristics and phenotype of MSCs

**Figure 1:**
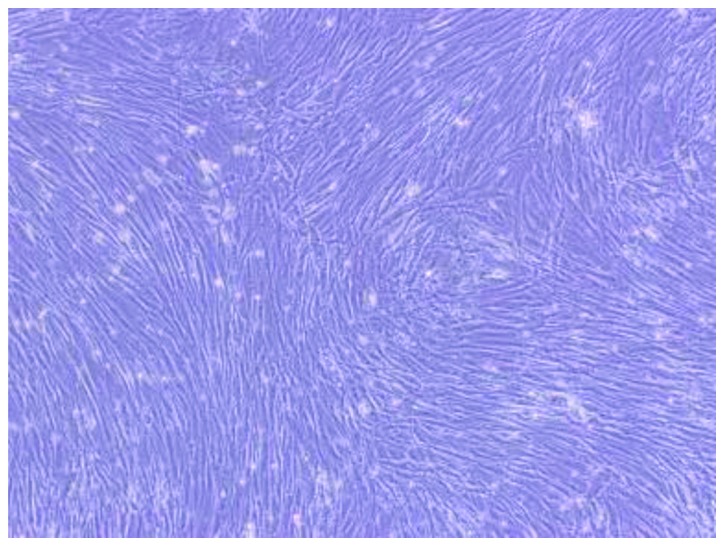
Human mesenchymal stem cells (MSCs) in monolayer culture. Inverted microscope images (magnification, ×100) showing MSCs in primary culture after 14 days.

The cells obtained from lymphocyte separation medium were round. Half of the growth media was replaced every 72 h. Single fusiform adherent cells and a few red cells were observed. With increased duration of culture, the number of adherent cells with long, fusiform cellular morphology increased rapidly. A few colonies of cells showed typical fibroblast morphology. The cells grew rapidly, reaching 70–80% confluence after 8–12 days. To expand the cultures, the monolayers were digested by trypsin and reseeded. These cells grew well and preserved the primary morphology of the parent cells (Fig. 1).

**Figure 2:**
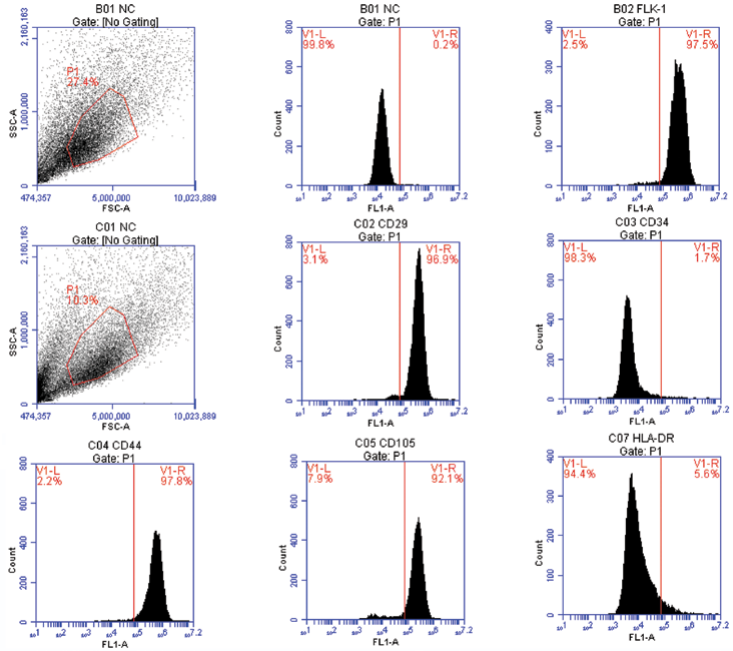
Phenotype analysis of the human mesenchymal stem cells.

As it is difficult to identify the MSCs using a simple cellular marker, the obtained cells were assessed by FCM (Fig. 2). The BMSCs uniformly expressed CD29, CD44 and Flk-1 and were negative for CD34 and HLA-DR.

### Effects of different dexamethasone concentrations on osteogenic capacity

**Figure 3:**
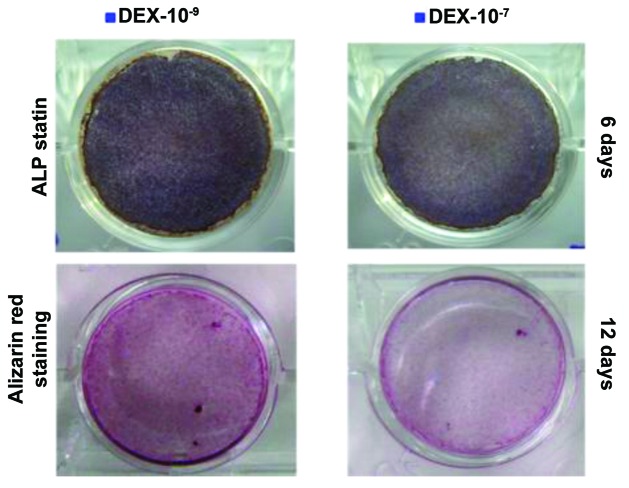
Osteogenic staining of mesenchymal stem cells showing the effect of dexamethasone on the osteogenic calcification of cells.

The staining for alkaline phosphatase and alizarin red showed positive results. In particular, for the alkaline phosphatase staining, blue sediments were observed in the cells; for the alizarin red staining, mineralized nodules in the extracellular matrix were stained orange. The osteogenic capacity of 10^−9^ mol/l dexamethasone was stronger compared to that of 10^−7^ mol/l dexamethasone, as indicated by a significant increase in intracellular and extracellular mineralized nodules and more intense staining. The intensity of the staining increased with treatment time (Fig. 3).

### Differences in the expression of relevant osteogenesis genes

**Figure 4:**
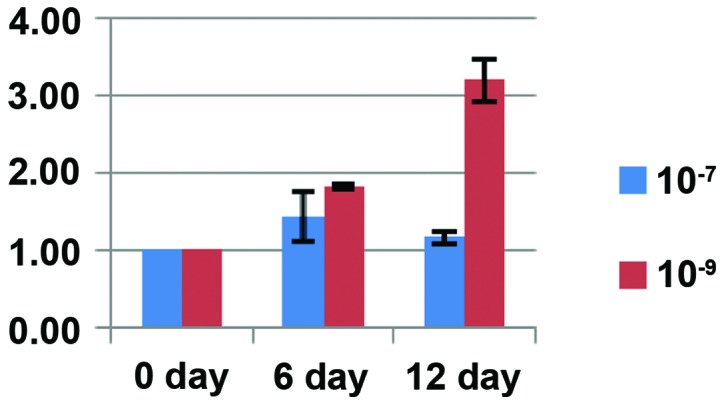
Osteocalcin expression following treatment of mesenchymal stem cells with 10^−7^ or 10^−9^ mol/l dexamethasone.

**Figure 5:**
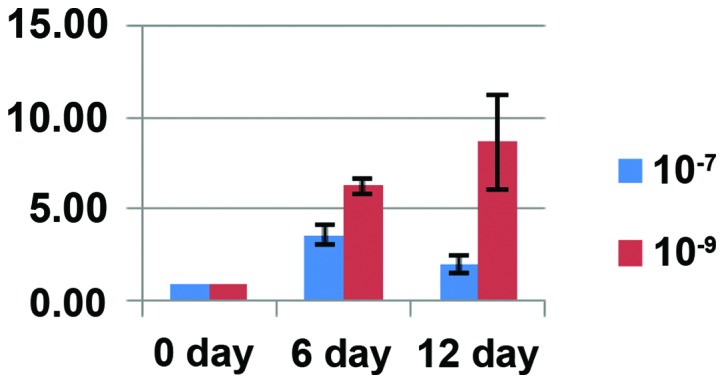
Osteopontin expression following treatment of mesenchymal stem cells with 10^−7^ or 10^−9^ mol/l dexamethasone.

**Figure 6:**
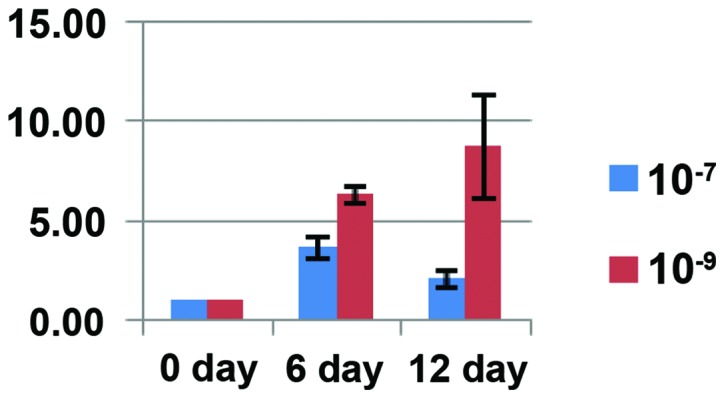
*Runx2* expression following treatment of mesenchymal stem cells with 10^−7^ or 10^−9^ mol/l dexamethasone.

The expression of the genes associated with osteogenesis, such as *OC*, *OPN* and *Runx2*, increased with time following dexamethasone induction. The expression of these genes was higher in the cells treated with 10^−9^ mol/l dexamethasone compared with the cells treated with 10^−7^ mol/l dexamethasone, reaching 2.73-, 4.34- and 2.36-fold higher expression, respectively. These results were statistically significant (P<0.05) (Figs. 4–6).

### High-throughput sequencing result and analysis

**Figure 7:**
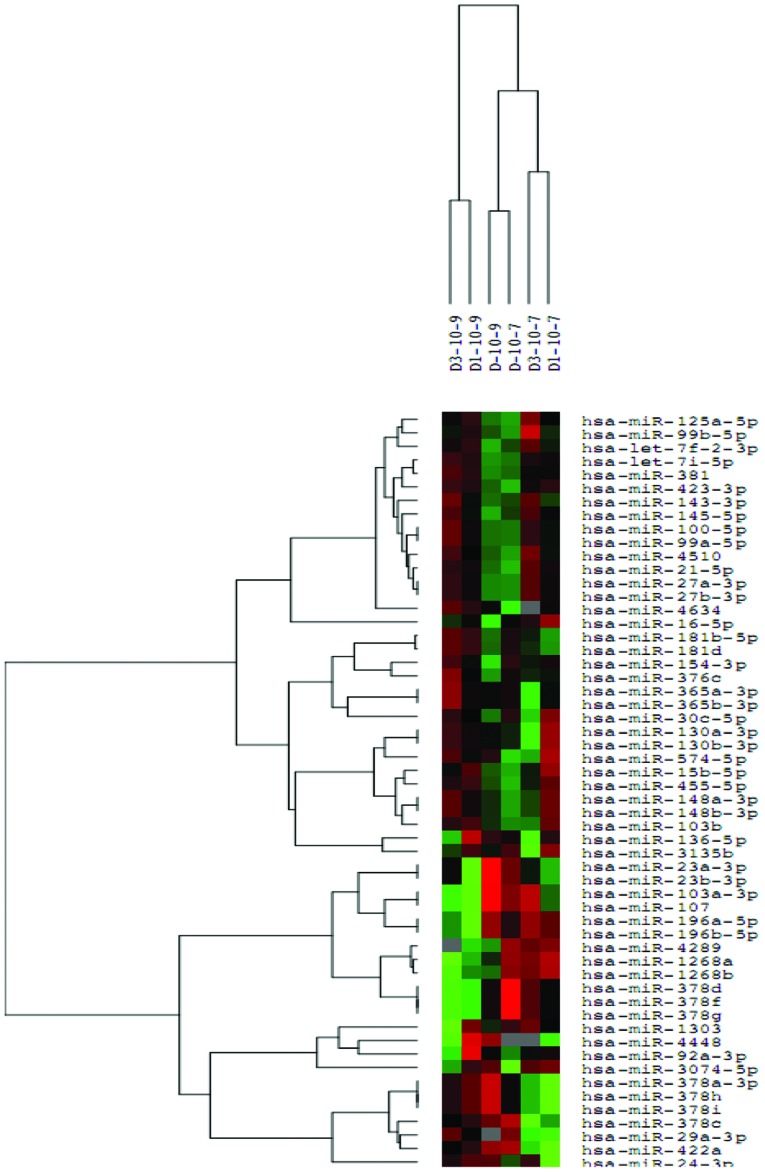
A heat map according to the statistical analysis. The green color represents the downregulation of microRNAs (miRNAs), and the red color represents the upregulation of miRNAs. The expression of miRNAs in the cells treated with 10^−7^ mol/l dexamethasone was different from that observed in the controls.

Following the normalization of the sequencing data, the miRNA expression differences (P<0.05) between the control cells and cells treated with 10^−7^ mol/l or 10^−9^ mol/l dexamethasone were assessed. The statistical significance between the groups was obtained from the triplicates of each group (Fig. 7).

The miRNA expression profile of MSCs was altered by treatment with 10^−9^ and 10^−7^ mol/l dexamethasone. A total of 16 miRNAs were consistently changed in the triplicates of each condition. Compared to treatment with 10^−9^ mol/l dexamethasone, 11 miRNAs were upregulated and 6 were downregulated following treatment with 10^−7^ mol/l dexamethasone (Table I).

## Discussion

miRNAs are highly conserved, small, single-stranded RNA molecules of 19–25 nucleotides that have been identified in eukaryotes. miRNAs have important roles in various cell functions and biological processes by triggering translation repression or the degradation of the target mRNA.

Currently, the study of miRNAs has received the most interest in tumor-related fields. For example, in 2002, abnormal miRNA expression was identified among a group of patients with chronic lymphocytic leukemia (CLL) characterized by a lack of 13q14 (17). Following this, numerous studies have reported the differential expression of miRNAs in cancer tissues. For example, the expression of the miRNA *let-7* family was reduced (18–20) in lung cancer. According to the study by Yu *et al* (21), the expression of *let-7* was higher in breast stem cells compared with breast cancer cells. In an *in vivo* xenograft model using breast cancer SK-3rd cells, cells over-expressing *let-7* grew more slowly and formed fewer tumors compared with the control cells. Using a miRNA microarray, Guo *et al* (22) identified that *miRNA-126* was absent in colon cancer and that *miRNA-126* had tumor-suppressive properties. The investigators also analyzed the miRNA expression profile of esophagus cancer tissues using the miRNA chip technique and identified 7 miRNAs (23) that could be used to distinguish normal and malignant tissues.

Recently, the number of studies on the role of miRNAs in MSC differentiation and regulation has been increasing. *miRNA-208* (24), *miRNA-141* and *miRNA-200a* (25) have been shown to contribute to the differentiation of osteogenic cells through the regulation of *BMP-2*, *miRNA-125b* and *BMP-4* (26). *PPARγ*, *Bambi* and *Criml* are downregulated by the BMP/Runx2 signaling pathway, and *miRNA-20* promotes the differentiation of MSCs towards the osteogenic fate (27) by inhibiting these genes. *miRNA-210* facilitates the differentiation of osteogenic cells by inhibiting the TGF-β/activin signal pathway (28). *miRNA-27* (29), *miRNA-29a* (30) and *miRNA-29b* (31) facilitate the differentiation of MSC to osteogenic cells through the Wnt signaling pathway. However, these studies have not explored the spontaneous regulatory pathways that are present in stem cells, and there are still no definite reports regarding miRNA expression changes in stem cells under pathological conditions, such as steroid-induced femoral head necrosis.

In the present study, MSCs were obtained from 3 different individuals, and were separated and cultured *in vitro*. The differential expression of miRNAs was assessed by high-throughput sequencing following stimulation with different concentrations of dexamethasone. Subsequent to comparing and eliminating the differences among individuals, a large dose of glucocorticoids not only had a clear impact on the osteogenic differentiation of cells but also, compared to a normal physiological dose of dexamethasone, led to changes in the miRNA expression profile of MSCs.

Taken together with the results obtained in previous studies, the present study suggests that altered miRNA expression may have a role in the pathology of steroid-induced femoral head necrosis. Xia *et al* (32) studied the regulatory action of miRNAs in gastric cancer cell multidrug resistance and identified that the expression levels of miRNAs were altered in the MDR gastric cancer cells SGC7901/VCR. Specifically, the expression of *miR-15b* and *miR-16*, which belong to the *miR-15*/*16* family, were downregulated. However, *in vitro*, *miR-15b* and *miR-16* increased the sensitivity and reduced the resistance of SGC7901/VCR cells to chemotherapeutic drugs. In SGC7901/VCR cells, the study also reported that the Gcl-2 protein was upregulated and that the upregulation of *miR-15b* and *miR-16* reduced the luciferase activity of a reporter construct for B-cell lymphoma 2 (BCL-2), as well as the *Bcl-2* levels. These findings indicate that BCL-2 is the direct target of *miR-15b* and *miR-16*. Additionally, the expression of *miR-15b* and *miR-16* induced the apoptosis of SGC7901/VCR cells. This result indicates that *miR-15* and *miR-16* regulate BCL and its downstream signaling pathways. In the present study, *miR-16* expression was upregulated by a high-dose of dexamethasone.

The present study shows that *miRNA-103* and its homologous gene, *miRNA-107*, are highly conserved in vertebrates (33,34) and have a key role in fat metabolism. Xian-Zi *et al* (35) identified miRNAs that were associated with fatty acid metabolism and validated several target genes of miRNA that were involved in this process. Additionally, Joven *et al* (36) identified that polyphenols could prevent fatty liver disease induced by a high-fat diet in mice by modulating the expression of *miR-103*/*107*. Wilfred *et al* (37) reported the mechanism of miRNAs that regulate metabolism and suggested a possible action pathway of the *miRNA-103*/*107* intron region that influences the phosphorylation of pantothenic acid, an important rate-limiting step for generating coenzyme A. This rate influences several biochemical reactions involved in fatty acid metabolism, the tricarboxylic acid cycle and amino acid metabolism. It has been suggested that steroid-induced femoral head necrosis arises from disorders in lipid metabolism, and animal experiments (38,39) have shown that femoral head necrosis can arise following the administration of hormones and lipid-lowering drugs. These findings further support our hypothesis.

Kahai *et al* (40) reported that *miRNA-378* regulated the osteogenic differentiation of cells through nephronectin (NPNT), an extracellular matrix protein. The expression of *NPNT* is extremely high, and the stable transfection of MCST3-E1 primary mouse osteogenic cells with *miRNA-378* altered the *NPNT* levels. Of note, the present study identified that the subfamily members of *miRNA-378* are not only upregulated but are also downregulated following treatment with high concentrations of dexamethasone. This result may have been due to the sensitivity of the high-throughput sequencing methods. However, the specific mechanisms of action of *miRNA-378* require elucidation in future studies.

miRNAs have an important regulatory role in the osteogenic differentiation of MSCs. The present study shows that high concentrations of glucocorticosteroids can result in changes in the miRNA expression profile of MSCs and this change may regulate the pathophysiological process of steroid-induced femoral head necrosis. The miRNA expression analysis provides a primary physiopathological mechanism that accounts for the steroid-induced ANFH. Additionally, this analysis offers novel treatment methods and the potential for the development of early intervention and stem cell therapy methods.

## Figures and Tables

**Table I:** Changes in the miRNA expression profile of human mesenchymal stem cells following treatment with 10^−7^ or 10^−9^ mol/l dexamethasone.

Change in expression	miRNA
Upregulated	*hsa-miR-4289*
	*hsa-miR-378g*
	*hsa-miR-378f*
	*hsa-miR-378d*
	*hsa-miR-196b-5p*
	*hsa-miR-196a-5p*
	*hsa-miR-16-5p*
	*hsa-miR-1268b*
	*hsa-miR-1268a*
	*hsa-miR-107*
	*hsa-miR-103a-3p*
Downregulated	*hsa-miR-4634*
	*hsa-miR-4448*
	*hsa-miR-378i*
	*hsa-miR-378h*
	*hsa-miR-378a-3p*
	*hsa-miR-24-3p*
